# Tunable Hypersonic Resonators via Electron‐Irradiation‐Induced Giant Modulation of Microparticle Elasticity

**DOI:** 10.1002/smll.202410278

**Published:** 2025-03-24

**Authors:** Francesco Bonacci, Francesco Cottone, Alessandro Di Michele, Alessandra Anna Passeri, Marco Madami, Silvia Caponi, Maurizio Mattarelli

**Affiliations:** ^1^ Dipartimento di Fisica e Geologia Università di Perugia Via A. Pascoli Perugia 06123 Italy; ^2^ Istituto Nazionale di Fisica Nucleare ‐ Sez. di Perugia Via A. Pascoli Perugia 06123 Italy; ^3^ CNR ‐ Istituto Officina dei Materiali (IOM) Unità di Perugia Via A. Pascoli Perugia 06123 Italy

**Keywords:** acoustic resonators, brillouin light scattering, irradiation‐assisted nano‐manipulation, phononic nanomaterials, stöber microparticles

## Abstract

The ability to modulate the elastic properties of nanostructured objects is crucial for the development of innovative materials able to control the propagation of acoustic waves (phonons) in the hypersonic frequency regime, with applications ranging from acousto‐ to thermo‐optical devices. Here, an advanced strategy is explored to finely tune the elastic properties of Stöber silica microparticles, commonly used building blocks for phononic materials. Using moderate electron beam energies in a scanning electron microscope, a controlled, huge and rapid particle elasticity tuning is demonstrated, which is investigated by Brillouin light scattering. The findings are interpreted in terms of an irradiation‐induced stiffening of the contacts between the primary nanoparticles composing the Stöber particle, attributable to changes in the silica network through radiolytic processes. The versatile control of the mechanical properties of microparticles, combined with their electret‐like behavior upon charging, offers broad‐spectrum possibilities for coupling phononic properties with external electromagnetic fields, paving the way for innovative phononic materials.

## Graphical abstract

A strategy is presented to tune the elastic properties of Stöber silica microparticles, widely used in phononic materials, by exposing them to moderate electron beam energies. This results in rapid elasticity changes, investigated via Brillouin light scattering, and attributed to radiation‐induced stiffening. This approach offers new possibilities for coupling phononic properties with electromagnetic fields in advanced materials.

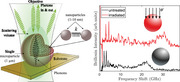

## 1 Introduction

Hypersonic phononic crystals (PnCs) and metamaterials promise groundbreaking advancements in the manipulation of high‐frequency vibrations, with applications ranging from tunable acoustic filters^[^ ^1^ ^]^ to optomechanical devices^[^ ^2^ ^]^ and heat management at the nanoscale.^[^ ^3^ ^]^ In recent years, the research on these materials has gained renewed momentum thanks to the use of local resonances as an additional mechanism for controlling hypersounds.^[^ ^4^ ^]^ For instance, coupling effective‐medium acoustic waves with local resonant modes in phononic crystals creates hybridization bandgaps (HGs),^[^ ^5^, ^6^ ^]^ often robust to structural imperfections. Various strategies have been explored to create HGs in the sub and super‐wavelength regimes through manipulation of confined resonances. Typical examples include sintering of nanoparticles in 3D PnCs,^[^ ^7^ ^]^ tuning the elastic impedance between particles and host material,^[^ ^8^ ^]^ changing the particle‐membrane adhesion in 2D PnCs,^[^ ^9^ ^]^ or introducing elastic anisotropy across the particle–polymer interface in composite materials.^[^ ^10^ ^]^ However, expanding the range of working frequencies remains challenging, due to the lack of experimental systems with variability in geometric and elastic properties, or the unavoidable large energy dissipation in bulk samples.^[^ ^11^ ^]^ While changing the resonator size is often used to control its characteristic eigenfrequencies,^[^ ^12^ ^]^ and hence the hybridization band structure, this also implies the modification of the crystal lattice spacing and associated interference‐induced Bragg‐like bandgaps, ultimately limiting the multiband filtering versatility of the material.

In this work, we provide guidelines for precisely and selectively tuning the resonance properties of typical building blocks for phononic materials, specifically silica microparticles made by the Stöber process, which does not involve changing the typical resonator size or composition. Our approach is based on an ultra‐fast, precise and significant modulation of the particle elasticity, and associated vibrational eigenfrequencies, which only requires relatively short low‐energy irradiation by the electron‐beam of a Scanning Electron Microscope (SEM). By varying the electron beam energy, we not only modulate the charging state of the particles,^[^ ^13^ ^]^ but also achieve a controllable and significant material stiffening, which we attribute to the radiolytic formation of defects at the nanocontacts between the primary particles comprising the porous Stöber beads. This change in rigidity is monitored using Brillouin Light Scattering (BLS), a spectroscopic technique based on the frequency analysis of inelastically scattered light, which, compared to other methods such as Atomic Force Microscopy or nano indenters,^[^ ^14^ ^]^ has the advantage of being fully contactless, thereby avoiding alteration in the injected charge and providing the bulk properties.

Until now, the mechanical strength of silica microparticles has primarily been altered through thermal treatments,^[^ ^15^, ^16^ ^]^ which may face limitations when applied to phononic materials, especially when selective modification of the mechanical properties of individual particles or clusters after material assembly is needed. Moreover, thermal and pressure treatments are difficult to control with sub‐micron precision. The proposed method overcomes these challenges. First, the ability to focus the electron beam into nanometer‐sized spots and adjust the interaction volume by varying beam energy enables precise tailoring of both mechanical and electronic properties in three dimensions at the nanometer scale. Second, this approach is compatible with post‐assembly modification, a crucial advantage for colloidal‐based phononic materials fabricated using bottom‐up techniques. Lastly, the method is efficient and potentially scalable, allowing for the treatment of a large number of structures within a reasonable timeframe.

## 2 Results and Discussion

### 2.1 Stiffness Measurement of Single Microparticles Using BLS Experiments

**Figure 1:**
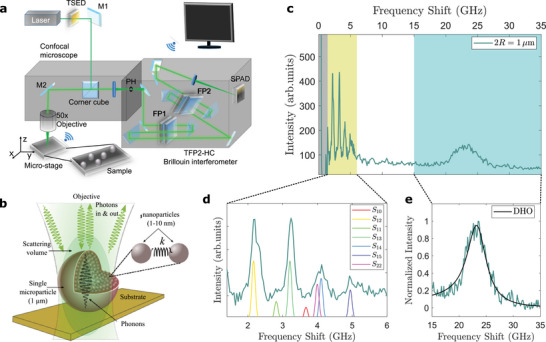
a) Sketch of the experimental apparatus used to measure the acoustic properties of individual particles. Temperature stabilized etalon device (TSED), single photon avalanche detector (SPAD), Fabry‐Pérot interferometers (FP), mirrors (M), and pinhole (PH) are represented (see the Experimental Section). b) The Stöber particle is formed through aggregation of primary nanoparticles of bond stiffness *k*. c) A typical Brillouin spectrum of a 1 µm‐sized particle, showing the longitudinal peak at high frequencies and confined Lamb modes below 10 GHz. d) Experimental and reconstructed spheroidal (*S*_*n*ℓ_) Lamb modes. e) Zoom on the longitudinal peak, fitted to a damped harmonic oscillator (DHO) function (see the Experimental Section).

The mechanical properties of the particles are studied using an innovative Brillouin microscope operating in backscattering geometry,^[^ ^17^ ^]^ see **Figure** **1**a. It is based on a single‐mode laser working at wavelength λ = 532 nm, coupled with a custom confocal microscope and a high‐contrast tandem Fabry‐Perot interferometer for the analysis of the backscattered light (refer to ref. [17] and Experimental Section for a more detailed description). The unparalleled contrast and high resolution in both the spatial and frequency domains achieved by our configuration enable the detection of tiny spectroscopic features, otherwise undetectable by any other setup. This capability is harnessed in this work to precisely characterize the stiffness of isolated microparticles. As far as we know, this study is the first to report the measurement of longitudinal and shear moduli of individual microparticles from BLS. As an example, we report in Figure 1b typical Brillouin data acquired on a particle with diameter of 1 µm. In the high frequency region, the spectrum exhibits a broad peak centered at ≈23 GHz (Figure 1c), that is assigned to the longitudinal acoustic mode producing a density modulation along the microparticle. The peak position, ν_*B*_ = *qv*_*L*_/2π, depends on the longitudinal sound velocity *v*_*L*_ and on the scattering wavevector *q* = 4*n*π/λsin (θ/2), where *n* is the material refractive index and θ the angle between the incident and scattered light (θ ∼ π in backscattering configuration). A second distinct mechanism of vibration produces sharp peaks that can be discriminated in the low frequency region of the spectrum (below 10 GHz). They are related to spontaneous vibrations of the whole particle that induce a volume variation and a consequent modulation of the scattered light. The frequency position of these quantized modes can be described using the theory formulated by Lamb for the free vibration of a homogeneous elastic spheres of radius *R*:

(1) $$S_{n\ell }=\xi_{n\ell }\left(v_{L},v_{T}\right)\frac{v_{L}}{R}$$

where the coefficient ξ_*nl*_, which depends on both the longitudinal and the transverse velocity, *v*_*T*_, indicates a vibrational mode with (*n* − 1) radial nodes and 2*l* surface ones.^[^ ^18^ ^]^ Note that only spheroidal modes *S* are considered in the spectrum, as torsional modes can not be observed due to selection rules for light scattering.^[^ ^18^ ^]^ The approximation of a freely vibrating sphere is fully justified in our case, as the effect of substrate contact is weak for our relatively large particles.^[^ ^19^ ^]^ Any influence from the substrate should manifest only as a slight change in peak linewidth, which, in our case, is primarily governed by instrumental resolution (100 MHz).

The simultaneous and independent measurement of bulk and confined modes of the individual particle allows for its precise mechanical characterization. First, from the position of the high frequency peak (Figure 1c), *v*_*L*_ and thus the longitudinal elastic modulus $M=\rho v_{L}^{2}$ of the particle can be estimated, knowing the density ρ. Thereafter, the previously determined *v*_*L*_ and the measured position of the Lamb peaks can be used to determine the transverse sound velocity *v*_*T*_ and particle shear modulus $G=\rho v_{T}^{2}$, as illustrated in Figure 1d (see also Experimental Section). The unique capability of detecting the Brillouin signal from a single particle offers a distinct advantage over previous studies conducted on packed or loose particle aggregates.^[^ ^18^, ^20^ ^]^ In aggregates, while the high signal from the longitudinal peak is beneficial, it comes at the expense of a reduced definition in *q*, due to multiple light scattering. This uncertainty transfers to *v*_*L*_ and, in turn, to the longitudinal elastic modulus estimate. On the other hand, although the position of the Lamb modes does not depend on *q*, which only affects their intensity, measuring a single particle, with precisely known radius (see Supporting Information), has the advantage of removing the heterogeneous peak broadening related to polydispersity in aggregates (Equation 1).

### 2.2 Stöber Particle Elasticity Depends on their Internal Granular Structure

In the case presented in Figure 1, our analysis provides *v*_*L*_ ≈ 4.48 km s^−1^ and *v*_*T*_ ≈ 2.57 km s^−1^. These values are comparable with those previously observed on similarly prepared SiO_2_ particles.^[^ ^18^, ^20^ ^]^ Using the density given by the manufacturer, ρ = 1.96 g cm^−3^, we obtain *M* = 39 GPa and *G* = 13 GPa, indicating that the studied particles are much softer than bulk silica (*M*_*B*_ = 77 and *G*_*B*_ = 30 GPa^[^ ^21^ ^]^), in agreement with previous studies.^[^ ^14^ ^]^ This observation is only partially ascribed to the particle porosity. Considering the linear relationship between sound velocity and density,^[^ ^22^ ^]^ this would lead to *v*_*L*_ ≈ 5.2 km s^−1^, well above the measured values. We relate the additional effect to the inhomogeneous nature of the studied particles. Stöber growth of large SiO_2_ beads is a multiple stage process with subsequent depositions of small (5 − 10 nm) primary particles on a seed.^[^ ^23^ ^]^ Although the particle can be still considered as elastically homogeneous in our analysis (the average pore size, 3 − 10 nm, is much smaller than the phonon wavelength Λ = λ/2*n* ≈ 190 nm), this aggregation process introduces a large amount of mechanical interfaces, which influences the propagation of acoustic vibrations and cause a decrease of phonon speed, as observed.

The granular appearance of the studied particles suggests that their elasticity is also determined by the stiffness *k* of the nanocontacts between the primary particles, as illustrated in Figure 1b. Consequently, altering the overall mechanical properties of the particles could be achieved by directly modifying *k*, as is commonly done in granular aggregates through methods such as polymerization,^[^ ^24^ ^]^ pressure application^[^ ^25^ ^]^ or thermal annealing.^[^ ^19^ ^]^ In the following, by leveraging the unique structure of the studied particles and the fine spatial control provided by the SEM electron beam, we demonstrate how to fabricate elastically tunable acoustic resonators by locally modifying the bond stiffness at the nanocontacts.

### 2.3 Tuning Particle Stiffness via Low‐Energy Electron Irradiation

**Figure 2:**
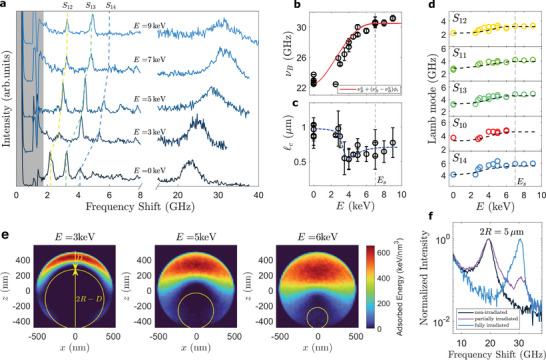
a) Brillouin spectra of 1 µm particles irradiated at different electron beam energies *E*. b) Longitudinal peak frequency as a function of *E*. Red line is the prediction of Equations (2) and (3). c) Phonon coherence length versus *E*. Dashed blue line is a guide for the eye. d) Spheroidal *S*_*n*ℓ_ Lamb modes versus *E*. Dashed lines correspond to a finite element analysis based on the same model (Experimental section). e) Monte Carlo simulations of the total absorbed energy during electron beam scanning at various *E*. f) Brillouin spectrum acquired on a partially irradiated 5 µm particle, showing both the Brillouin peaks of the unirradiated (18.5 GHz) and irradiated (30.5 GHz) domains.

**Figure** **2**a showcases the dramatic effect of the irradiation on the Brillouin signal acquired from various 1 µm particles exposed to different beam energies in the range *E* = 2.5 − 9 keV. A significant blue shift is observed in both the longitudinal peak and Lamb modes as *E* increases. In particular, the longitudinal peak increases from $\nu_{B}^{u}\approx \;23$ GHz for unirradiated particles to $\nu_{B}^{i}\approx \;30.5$ GHz for fully irradiated particles (Figure 2b). A comparable increase is observed across all spheroidal modes *S*_*n*ℓ_ (Figure 2d). Taken together, these data suggest a significant stiffening of the studied particles upon irradiation.

To shed light on the observed behavior, we conducted Monte Carlo simulations using the free software package CASINO, version 3.3^[^ ^26^ ^]^ (Experimental Section). We quantify the effect of radiation energy and dose on the sample mechanical properties by computing the spatial distribution of deposited energy within the particle. The simulation results are presented in Figure 2e, where the absorbed energy distribution along a vertical plane passing through the particle center, is shown for various *E*. Our simulations reveal that energy deposition primarily occurs within a hollow spherical cap region located at the top of the particle, with the size of this region depending on *E*. This behavior can be attributed to the electron penetration depth, *D*∝*E* ^1.67^,^[^ ^27^ ^]^ which represents the depth at which the transmitted electrons' average energy vanishes, and the effective sample thickness seen by the incident electrons. In fact, both the effective thickness and *D* decrease with the angle of incidence between the impinging electrons and the particle surface,^[^ ^28^ ^]^ resulting in a reduction of the irradiated volume as the beam moves from the center to the particle periphery. Additionally, as the scanning extends slightly beyond the particle boundary, backscattered electrons (BSEs) emerging from the substrate can strike the particle from below at energies comparable to the primary electrons. This phenomenon can explain the observed energy deposition at the particle's bottom, even at low *E*.

Based on these insights, we model the irradiated volume, Ω_*i*_, by representing it as the negative of a spherical unirradiated region of size 2*R* − *D* located at the bottom of the particle, as depicted in Figure 2e. Ω_*i*_ increases with the primary beam energy, as expected, and importantly, reaches the entire particle volume Ω_*p*_ when *D* equals the particle diameter. This occurs at around *E*_*s*_ ≈ 7 keV, which also corresponds to the saturation of the Brillouin peak (Figure 2b). This last observation suggests that the particle stiffening may be described, in a fist approximation, on the basis of *D*, by considering the relative volumes of the irradiated and non‐irradiated regions. Specifically, we propose modeling the Brillouin data by assuming that electron bombardment only modifies the mechanical properties of the irradiated volume while leaving the non‐irradiated region unaffected. To demonstrate this, we first compute the irradiated volume fraction ϕ_*i*_ = Ω_*i*_/Ω_*p*_ as:

(2) $$\phi_{i}=\left\{\begin{array}{c} 1-{\left(1-D/(2R)\right)}^{3}\;\;\;\text{if}\;D<2R,\\ 1\;\;\;\;\text{if}\;D\geq 2R \end{array}\right.$$

Then, considering that the phonons extend through all the samples in the absence of sharp interfaces, we describe the measured ν_*B*_ as an average between the volume of the (stiffer) irradiated region and that of the (softer) unexposed one:

(3) $$\nu_{B}=\nu_{B}^{u}+\left(\nu_{B}^{i}-\nu_{B}^{u}\right)\phi_{i}$$

Note that, in our experimental conditions, no significant change of the refractive index is expected,^[^ ^29^ ^]^ even for the fully irradiated particle, therefore the blue shift of ν_*B*_ provides directly the increase of *v*_*L*_ and material rigidity. The predictions of Equations (2) and (3) are reported in Figure 2b, showing a remarkable agreement with the experimental peak positions, especially for energies above 3 – 4 keV. Below *E* ≈ 2.5 keV, the electron penetration depth is shallow (<150 nm), meaning that most of the energy is deposited near the surface of the particle. The corresponding irradiated volume is sufficiently small that its contribution to the whole mechanical properties of the particle, and hence to the Brillouin signal, is hardly detectable.

As a matter of fact, our Brillouin measurements cannot distinguish the contributions coming from the two regions with different mechanical properties (Figure 2a). The presence of a single longitudinal peak is not surprising, however. It confirms a fundamental property of the longitudinal acoustic modes: they can be sustained by a single material only when its characteristic size is greater than ≈3 phonon wavelengths.^[^ ^20^ ^]^ This condition sets an effective cutoff length δ_co_ ≈ 3Λ ≈ 0.5 µm for the detection of the acoustic modes: when *D* or 2*R* − *D* ⩽ δ_co_, only one single peak is expected in the spectrum and its frequency shift is related to the mechanical properties averaged over the phonon coherence length, ℓ_*c*_, the characteristic size over which the density fluctuations in BLS process are correlated.^[^ ^30^ ^]^ The phonon coherence length can be directly determined from the Brillouin spectrum, ℓ_*c*_ = Λν_*B*_/Γ_*B*_, where Γ_*B*_ is the peak width, and the obtained values as a function of *E* are reported in Figure 2c. For the untreated 1 µm particles, ℓ_*c*_ is principally determined by the confinement effect (ℓ_*c*_ ≲ 0.95 µm). The non‐uniform irradiation further reduces ℓ_*c*_: as illustrated, the coherence length exhibits a minimum (ℓ_*c*_ ∼ *R* = 0.5 µm) when the interaction volume is approximately half of the particle volume (ϕ_*i*_ ∼ 0.5, *E* = 4 keV), before eventually increasing again as the particle becomes more uniformly irradiated for larger *E*.

To further validate our interpretation, we examined larger particles with diameter of 2*R* = 5 µm, for which the characteristic size of the non‐irradiated and irradiated regions can be made large enough to exceed the cut‐off length δ_co_, when SEM exposure is carried out at intermediate penetration depths. As shown in Figure 2f, spectra acquired from these particles indeed display two distinct Brillouin peaks, with frequency shifts corresponding to the different mechanical properties of the irradiated and unexposed domains. Additionally, the linewidth analysis in these larger untreated particles reveals a coherence length ℓ_*c*_ = 1.1 − 1.5 µm: even when the confinement effect is removed, the presence of mechanical heterogeneities results in a phonon coherence length much smaller than the value found in bulk silica, where ℓ_*c*_ is tens of microns^[^ ^22^ ^]^—providing additional evidence of the mechanical heterogeneity introduced by the Stöber process.

**Figure 3:**
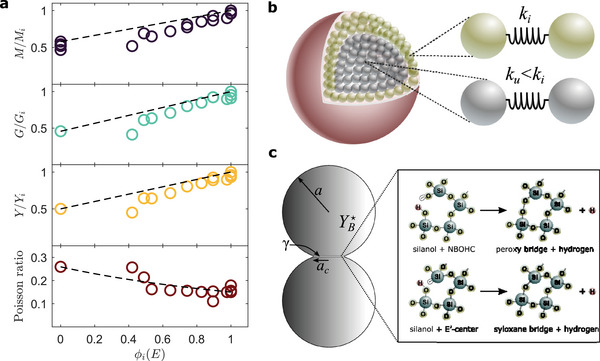
a) Normalized longitudinal, shear, Young's modulus, and Poisson ratio as a function of the irradiated volume ratio. Dashed lines are from Equations (2) and (3). b) The observed stiffening is attributed to an increase of the primary particle bond stiffness *k*_*i*_ > *k*_*u*_ in the irradiated region. c) Possible structural changes in the contact zone induced by the electron bombardment, involving the release of hydrogen in atomic form. In the NBOHC and E′‐center native defects, the arrow denotes the unpaired electron and the dashed balloon its orbital.

Finally, a further confirmation of the proposed stiffening model comes from finite element calculations of the Lamb vibrations, assuming the particle is composed of two distinct elastic domains (see Experimental Section). Figure 2d demonstrates the excellent agreement between the calculated and experimental frequencies for modes with *n* = 1 and *l* = 0 − 4, showed in order of increasing vibrational energy. Hence, by comparing the measured eigenfrequencies with those of elastically homogeneous spheres, we obtain an estimate of *G*, which, together with *M* and the hypothesis of isotropic linear elasticity, provides a complete description of the elastic properties of our particles (**Figure** **3**a). Interestingly, the maximum values obtained for fully irradiated particles are close to those found for fused silica,^[^ ^21^ ^]^ corresponding to a remarkable ∼100% increase of the elastic moduli (from *M*_*u*_ = 39 and *G*_*u*_ = 13 GPa to *M*_*i*_ = 69 and *G*_*i*_ = 28 GPa).

### 2.4 Electron Irradiation Modifies Contact Stiffness at Internal “Grain” Boundaries

SEM irradiation has proven to be a highly effective tool for increasing the stiffness of our particles. The above discussion shows that this stiffening can be effectively explained only on the basis of the penetration depth of the primary electrons. In a previous work,^[^ ^13^ ^]^ we demonstrated that our Stöber microparticles exposed to similar beam energies, also become significantly charged due to electron trapping within the particle. Although particle charging can be explained using similar arguments based on the penetration depth of the primary electrons,^[^ ^13^ ^]^ it is important to note that the huge increase in rigidity we observed here cannot be ascribed to a direct effect of trapped charges. First, as the charge density is a volume property, its change should only affect the longitudinal dynamics associated to density fluctuations (pressure waves). In contrast, we find that the changes in both longitudinal and shear moduli are comparable. Second, despite conducting BLS experiments immediately after SEM exposure, charge neutralization due to ions naturally present in the air can not be avoided. In ref. [13], we demonstrated that charge decay in Stöber microparticles occurs over two characteristic time constants: a rapid one, lasting several hours, associated with surface discharge, and a slower one, extending over several months, related to inner charge diffusion. Consequently, we expect charge decay to be more significant in particles charged only on the surface, such as those exposed to low beam energies.^[^ ^13^ ^]^ However, this effect is not clearly reflected in our Brillouin data. Additionally, if particle stiffening were due solely to charge accumulation, we would expect a marked reduction in elasticity as the particles lose charge. We monitored the shift in the longitudinal peak of charged particles stored in ambient conditions over several days (Figure S2, Supporting Information), yet no clear effect from charge decay was observed.

The observed huge and stable particle stiffening, therefore, must arise from changes in the atomic network structure, i.e., beam damage, induced by electron irradiation. We anticipate that these structural modifications mainly occur in the contact region between the primary nanoparticles. In fact, the beam energies used in our study are not sufficient to displace an Si and/or an O atom from its lattice site to an interstitial position from direct transfer of momentum (knock‐on process). We thus expect negligible radiation‐induced modifications to the bulk SiO_2_ network of the primary nanoparticles, consistently with the observation that the measured longitudinal sound velocity grows with irradiation till reaching a value comparable with that of pure SiO_2_. On the contrary, the beam energies explored in this study are known to induce radiolytic processes, wherein atomic displacements and bond breaking occur through the nonradiative relaxation of electronic excitations.^[^ ^31^, ^32^ ^]^ Defects formed radiolytically are more likely localized at grain boundaries,^[^ ^33^, ^34^ ^]^ where the densities of defect precursor, strained bonds, and impurities (e.g. silanols in highly hydrated silica, like our Stöber particles^[^ ^15^ ^]^), are the highest.^[^ ^32^, ^35^ ^]^ Radiolysis of the O–H bond, in particular, releases hydrogen in atomic form and produces nonbridging oxygen hole centers (NBOHC), i.e. silanol radicals,^[^ ^36^, ^37^ ^]^ This process is eventually accompanied by the desorption of hydrogen molecules through electromigration by the strong electric fields from trapped charges. Radiolytically produced O_2_ desorption is also possible.^[^ ^35^ ^]^ Silanol radicals are active species that can produce stable peroxy siloxanes^[^ ^37^ ^]^ or recombine with E′‐centers (silicon dangling bonds with an unpaired electron) to form bridging Si–O–Si bonds,^[^ ^38^ ^]^ as illustrated in Figure 3c. When these reactions occur on particle surfaces, they are known to influence the binding strength between particles.^[^ ^39^ ^]^ These processes may be promoted by the potential temperature rise during electron bombardment,^[^ ^40^ ^]^ though significant heating is unlikely under our experimental conditions.^[^ ^41^ ^]^

In our Stöber particles, we argue that SEM exposure increases the number of crosslinks between primary particles, resulting in local densification and overall stiffening of the silica network in this region. In light of this, we rationalize our observations by considering the particle as a disordered isotropic ensemble of nanospheres interconnected by cohesive interparticle bonds of stiffness *k*, whereby the effect of irradiation is to induce an increase in *k*, i.e. *k*_*i*_ > *k*_*u*_, see sketch in Figure 3b. For such systems, an expression of the Young's modulus has been given in ref. [42]: *Y* = 3 *k* *Z* (1 − ϵ)/(2π *a*), where *Z* is the mean number of bonds per (primary) particle, *a* their radius and ϵ the porosity. We use JKR theory for the contact stiffness of two adhesive spheres in the absence of external loads:^[^ ^43^ ^]^ $k=\left(6/5\right)\;Y_{B}^{★}a_{c}$, where $Y_{B}^{★}=Y_{B}/(2\;\left(1-\nu_{0}^{2}\right)≃\;37$ GPa is the reduced modulus of bulk silica and $a_{c}={\left(9\pi \;a^{2}\;\gamma /\left(4Y_{B}^{★}\right)\right)}^{1/3}$ the radius of the circular contact area, *γ* being the surface energy. By taking *a* = 5 nm and *γ* ≈ 50 mJ m^−2^ as typical values,^[^ ^23^, ^44^ ^]^ and considering a large *Z* (≈12) due to the small porosity, we get *Y*_*u*_ ≈ 30  GPa, in good agreement with the experimental value of 32.5 GPa of a non‐irradiated particle.

In this context, the *k*‐increase may be related to the growth of the neck *a*_*c*_ between the primary particles due to an increase in surface energy γ, resulting from the formation of siloxane bridges and local densification at interfaces, as discussed above (Figure 3b,c). The extrapolated maximum value of the surface energy γ_*i*_ = γ_*u*_ (*Y*_*i*_/*Y*_*u*_)^3^ ≈ 400 mJ m^−^^2^ is consistent with this picture. Indeed, for a fully siloxane surface, typically obtained by annealing above 2000 °C, *γ* ≈ 1000 mJ m^−2^,^[^ ^44^ ^]^ and the highest measured modulus is *Y* ≈ 85 GPa.^[^ ^21^ ^]^ This aligns well with our extrapolated highest modulus of 77 GPa, after accounting for a ≈10% effect due to porosity.

## 3 Conclusions

In conclusion, by using moderate electron‐beam irradiation in a SEM, we demonstrated a significant, precise (sub‐micron) and fast (few tens of seconds) modulation of the elastic and acoustic properties of Stöber silica microparticles. The pronounced effect we observed is attributed to the capability of the beam electrons to locally modify the bond stiffness between primary particles forming the studied particles. While the focus here was on Stöber silica, in situ modification of the interparticle stiffness by low‐energy particle irradiation is anticipated to be of great interest in other systems of high technological relevance.^[^ ^45^ ^]^ Potential candidates include nanocrystal (NC) solids, where collective NC vibrations are typically engineered through modification of the ligand elastic constant, via a careful design of their chemical composition and binding mechanism.^[^ ^46^ ^]^ Finally, our findings propose a novel avenue for external control of phononic materials. Simultaneous control of the charging state^[^ ^13^ ^]^ and elasticity enables the coupling of particle resonant modes with external electromagnetic fields. This advancement opens up exciting possibilities for the design of tunable phononic materials with remote control.

## 4 Experimental Section

### Sample Preparations

Water‐based suspensions of monodispersed Stöber silica microparticles, with a diameter of 1 and 5µm, were purchased from Sigma‐Aldrich and stored in the fridge. For the experiments, 50 µL of these solutions were diluted in 50 mL of pure ethanol (Fluka). Finally, the particles were deposited on a polished copper substrate by drop casting technique, i.e., by pouring a small drop of the diluted solution onto the substrate and letting evaporate at ambient temperature.

### SEM Characterization and Particle Irradiation

Particle visualization and irradiation was accomplished using a FE‐SEM LEO 1525 (ZEISS) with Gemini column equipped with a standard Everhart–Thornley (E–T) and an in‐lens detectors for the collection of secondary electrons (SEs). The instrument operates at high vacuum condition of about 2.5 × 10^−5^ mbar. Stiffening is achieved by irradiating the particles with electron beams at energies between 2 and 9 keV, using a slit aperture of 60 µm, corresponding to a current intensity, measured by a Faraday cup, in the range 0.4 – 0.7 nA.^[^ ^13^ ^]^

### Monte Carlo Simulations

To simulate the interaction between the electron beam and the silica particles, Monte Carlo simulations were performed using the version 3.3 of the free software CASINO.^[^ ^26^ ^]^ In alignment with the experimental setup, 1 µm‐sized SiO_2_ beads were placed onto a thick copper substrate and uniformly irradiated using a square raster region of 1095 nm side, slightly larger than the particle diameter. The region is divided into 5476 scan points separated by a step size of 15 nm. A Gaussian beam with diameter of 30 nm was used to guarantee overlap between points. These parameters have been chosen to reduce the computation time while preserving the number of electrons per pixel arriving to the sample, which is around 1000 considering our experimental conditions (*I* ∼ 0.4 nA, dwell time of τ ≈ 400 ns, pixel size of ≈2 nm). It was checked that using the exact scanning conditions does not change significantly the results. For each scan point, a Poisson distribution of the nominal number of electrons was used in order to simulate the effect of the electron gun shot noise. For the physical model implemented to calculate the electron trajectories, the interested reader can refer to ref. [47]. The deposited energy density in Figure 2e is computed by summing the energy loss for all trajectories and scan points in each unit volume.

### Brillouin Spectroscopy

Figure 1a illustrates the Brillouin microscope employed in this study, recently developed in the lab.^[^ ^17^ ^]^ In brief, a beam from a single‐mode solid‐state laser (SpectraPhysics Excelsior) emitting at λ = 532 nm is initially passed through a temperature‐controlled etalon (TSED TCF‐1, JRS Scientific Instruments, Tablestable Ltd., Mettmenstetten, Switzerland) to minimize secondary laser modes. The beam was subsequently focused onto the sample using a customized confocal microscope from JRS Scientific Instruments, which included a coaxial LED for illuminating and observing the sample surface. A three‐axis piezo translation stage, with a spatial resolution of approximately 0.01 µm, was used to locate isolated microparticles. The laser beam is focused on the particles by a long working distance microscope objective (Mitutoyo M‐Plan Apo 50×) with a numerical aperture (NA) of 0.42. The backscattered light was collected by the same objective, passed through a pinhole to reject out‐of‐focus scattered light, and was analyzed in frequency by a high‐contrast tandem Fabry‐Perot interferometer (TFP2 HC, JRS Scientific Instruments, Tablestable Ltd, Mettmenstetten, Switzerland), coupled with a single‐photon avalanche detector (SPAD, LaserComponents COUNT‐10). The TFP‐2 HC is a highly contrasted variant of the original Sandercock‐type 3+3 tandem multipass spectrometer, characterized by an unprecedented contrast of 150 dB and a high frequency resolution of 100 MHz. To resolve both high and low frequency peaks in the particle spectra, the free spectral range (FSR) of the spectrometer was adjusted by varying the mirror spacing from 3.5 to 12 mm. Further details of the instrumental setup can be found in ref. [17].

### Measurement of Individual Particle Sound Speeds

To access the particle longitudinal acoustic speed from the Brillouin peak ν_*B*_ = *qv*_*L*_/2π, first, the refractive index of the particle was computed from Lorentz–Lorenz relationship. Using ρ = 1.96 g cm^−3^ as provided by the manufacturer, *n* = 1.4 was obtained. This yielded an effective scattering wavevector equal to *q*_bs_ = 4π*n*/λ ∼ 0.033 nm^−1^. The high frequency peak was modeled using a damped harmonic oscillator function (DHO)

(4) $$I\left(\nu \right)=\frac{I_{0}}{\pi }\frac{\nu_{B}^{2}\Gamma_{B}}{{\left(\nu^{2}-\nu_{B}^{2}\right)}^{2}+\nu^{2}\Gamma_{B}^{2}},$$

with the peak width Γ_*B*_ = *D*_*k*_ *q* ^2^, where *D*_*k*_ is the kinematic viscosity, ν_*B*_ and *I*_0_ as free parameters. The fitting is performed considering the contributions of the *q*‐spread due to the objective lens, which amount to integrate the predicted DHO intensity from the minimum scattering wavevector *q*_min_ ≈ 0.975 *q*_bs_ to the maximum value *q*_bs_.^[^ ^48^ ^]^ The transverse acoustic speed of the particle was inferred from the analysis of the Lamb peaks using the obtained longitudinal speed and thanks to the prior knowledge of the particle radius *R*. The latter was measured with nanometer precision from SEM images, acquired before the charging process to avoid electrostatic aberrations,^[^ ^13^ ^]^ see also Supporting Information. As the relative separation between the different Lamb modes depended only on the ratio *v*_*L*_/*v*_*T*_,^[^ ^18^ ^]^ *v*_*T*_ is found by computing through Equation (1) the value that best matches all the experimental Lamb eigenfrequencies *S*_*n*ℓ_ identifiable in the spectra, as illustrated in Figure 1d. The relative contribution of each mode *S*_*n*ℓ_ to the total polarized Brillouin intensity was not calculated directly, as the computation was rather cumbersome and out of the scope of the present article. Interested readers are invited to refer to the method described in ref. [18].

### FEM Analysis

The finite element analysis was performed by using COMSOL multiphysics 5.0 with structural mechanics module. The particle was modeled with 2D axial symmetric geometry divided in two circular domains, representing the irradiated and untreated regions, with diameters or 2*R* and 2*R* − *D* respectively, where *D* represents the penetration depth. The latter is calculated using^[^ ^27^ ^]^

(5) $$D\left[\mu m\right]=\left(0.0276\cdot A/Z^{0.89}/\rho \right)E^{1.67}$$

where *A* and *Z* are respectively the atomic weight (in g mol^−1^) and atomic number of silica, ρ the density expressed in g cm^−3^ and *E* the beam energy in keV. The modal frequencies where computed by sweeping the penetration depth *D* while keeping constant the material density, Poisson ratio, shear and longitudinal elastic modulus, longitudinal and transversal sound velocity for each domain. These parameters were previously extrapolated from the Brillouin measurements, as described above, and given as input to the simulation software.

## Conflict of Interest

The authors declare no conflict of interest.

## Supporting information

Supporting Information

## Data Availability

The data that support the findings of this study are available from the corresponding author upon reasonable request.
