# Supplementary material for: Tunable Hypersonic Resonators via Electron‐Irradiation‐Induced Giant Modulation of Microparticle Elasticity
Source: Small. 2025 Mar 24;21(18):2410278. doi: 10.1002/smll.202410278 (PMC12051905; doi:10.1002/smll.202410278)
Supplement: Supplementary file 1 — Supporting Information [file SMLL-21-2410278-s001.pdf]

NANO · MICRO
small

Supporting Information

for *Small*, DOI 10.1002/smll.202410278

Tunable Hypersonic Resonators via Electron-Irradiation-Induced Giant Modulation of Microparticle Elasticity

Francesco Bonacci, Francesco Cottone, Alessandro Di Michele, Alessandra Anna Passeri, Marco Madami, Silvia Caponi* and Maurizio Mattarelli**

Supporting Information for "Tunable Hypersonic Resonators via Electron-Irradiation-Induced Giant Modulation of Microparticle Elasticity"

Francesco Bonacci[1,*], Francesco Cottone[1,3], Alessandro Di Michele[1], Alessandra Anna Passeri[1], Marco Madami[1], Silvia Caponi[2,*], Maurizio Mattarelli[1,*]

[1]**Dipartimento di Fisica e Geologia, Università di Perugia, Via A. Pascoli, 06123, Perugia, Italy**
[2]**CNR - Istituto Officina dei Materiali (IOM), Unità di Perugia, Via A. Pascoli, 06123, Perugia, Italy**
[3]**Istituto Nazionale di Fisica Nucleare - Sez. di Perugia, Via A. Pascoli, 06123, Perugia, Italy**
[*]**francesco.bonacci@unipg.it, silvia.caponi@cnr.it, maurizio.mattarelli@unipg.it**

Contents

1 Particle size measurements 1

2 Charge decay 1

1 Particle size measurements

The particle size is measured prior to the charging process to prevent electrostatic aberrations. Specifically, the development of a negative surface potential during charge accumulation can lead to a reduction in the apparent diameter, caused by the deflection of incoming electron trajectories toward the particle's periphery. This phenomenon can reduce the apparent size by as much as 10% [2], and has been utilized in the past to assess sample charging in much bigger particles [3]. To minimize charge buildup, we employ a low beam energy of 2 keV, which is close to the second crossover energy inferred from our SDHL data [4], where a global balance between incoming and emitted electrons is expected. The particle radius, R, is determined using a Circular Hough Transform (CHT) algorithm designed to detect circles in images [1], as illustrated in Figure S1. Table 1 presents the measured diameters for selected particles in this study, alongside the beam energies used for their successive charging. These measurements are subsequently applied in Equation 1 of the main text to calculate the transverse acoustic speed, v_T, based on the positions of the experimental Lamb modes, $S_{n\ell}$, and the value of v_L obtained from the longitudinal peak.

2 Charge decay

The particle stiffening observed after SEM exposure is not attributed solely to a direct effect of the charge accumulation. To demonstrate this, we tracked the position of the longitudinal peak of charged particles over several days, with the particles stored in ambient conditions (room temperature of 22 °C and humidity of $\sim$ 43%). As an example, Figure S2 presents the Brillouin peak of a 5 μm charged particle for three different time points after electron irradiation (E = 19 keV). As illustrated, the peak position of the longitudinal mode remains essentially constant for about three weeks, while we expected a significant charge decay over the same time window [4]. If particle stiffening were linked solely to the presence of trapped charges, we would expect the peak to shift toward lower frequencies over time. This observation signals the existence of other irreversible radiation-induced mechanisms by which the particles stiffen, as discussed in the main text.

Table 1: Particle size extracted from SEM image processing. The particles shown in Figure S1 correspond to those charged at 4.5 and 5 keV.

E (keV)	0	2.5	3	4.5	5	6	7	8	9
$2R$ (μm)	1.02	0.93	0.94	0.95	0.99	1.00	0.92	0.92	0.95

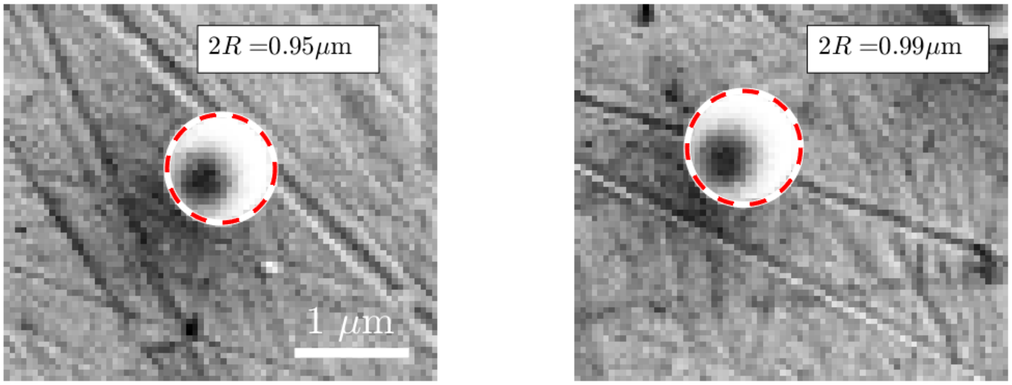

Figure S1: SEM images of silica particles prior to charging, acquired at 2 keV, in secondary electron mode. The dashed red lines are determined via a CHT algorithm [1], which also gives the radii of the particles.

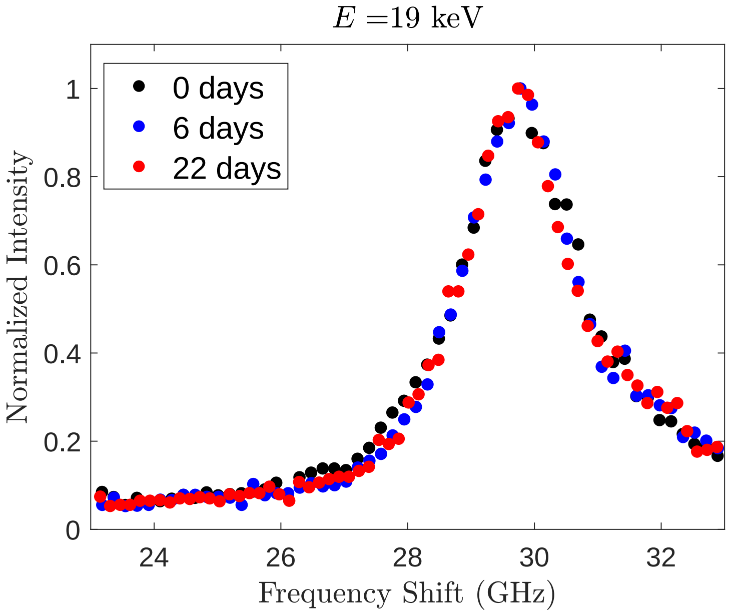

Figure S2: The longitudinal peak of a 5 μm particle charged at 19 keV, shown immediately after SEM irradiation, and at 6 and 22 days post-irradiation.

References

[1] H. Yuen, J. Princen, J. Illingworth, J. Kittler, *Image and Vision Computing* **1990**, *8*, 1 71.

[2] L. Reimer, *Scanning Electron Microscope-Physicis of Image Formation and Microanalysis*, volume 45, Springer-Verlag Berlin Heidelberg, **1998**.

[3] M. Belhaj, S. Odof, K. Msellak, O. Jbara, *Journal of Applied Physics* **2000**, *88*, 5 2289.

[4] F. Bonacci, A. Di Michele, S. Caponi, F. Cottone, M. Mattarelli, *Smart Materials and Structures* **2018**, *27*, 7.
